# Direct imaging of glymphatic transport using H_2_^17^O MRI

**DOI:** 10.1172/jci.insight.141159

**Published:** 2021-05-24

**Authors:** Mohammed S. Alshuhri, Lindsay Gallagher, Lorraine M. Work, William M. Holmes

**Affiliations:** 1 Institute of Neuroscience and Psychology, College of Medicine, Veterinary and Life Science, University of Glasgow, Glasgow, United Kingdom.; 2 Radiology and Medical Imaging Department, College of Applied Medical Sciences, Prince Sattam bin Abdulaziz University, Al Kharj, Saudi Arabia.; 3 Institute of Cardiovascular and Medical Sciences, College of Medicine, Veterinary and Life Science, University of Glasgow, Glasgow, United Kingdom.

**Keywords:** Development, Neuroscience, Neuroimaging, Neurological disorders, Transport

## Abstract

The recently proposed glymphatic pathway for solute transport and waste clearance from the brain has been the focus of intense debate. By exploiting an isotopically enriched MRI tracer, H_2_^17^O, we directly imaged glymphatic water transport in the rat brain in vivo. Our results reveal glymphatic transport that is dramatically faster and more extensive than previously thought and unlikely to be explained by diffusion alone. Moreover, we confirm the critical role of aquaporin-4 channels in glymphatic transport.

## Introduction

The transport of solutes within the brain parenchyma is of fundamental importance to nutrient delivery and the clearance of metabolites, neurotransmitters, and toxic macromolecules (e.g., β-amyloid). Conventionally, interstitial solutes were considered to be transported via diffusion, but recent evidence suggests an additional bulk flow of the interstitial fluid (ISF). The glymphatic (glial-lymphatic) hypothesis proposes that subarachnoid cerebrospinal fluid (CSF) is driven by arterial pulsation along the perivascular space surrounding penetrating arteries, with influx into the brain interstitium mediated by the astroglial water channel aquaporin-4 (AQP4) (1). It is proposed that this influx results in the bulk flow of ISF, which then exits along perivenous spaces, providing an efficient clearance mechanism for waste products from the parenchyma. Glymphatic transport has been shown to increase during sleep, and this increases the clearance of β-amyloid in mice, potentially explaining the decades old mystery of why sleep is restorative (2). Glymphatic transport has been further shown to increase in ischemic stroke and play an important role in poststroke edema (3). Conversely, decreased glymphatic transport has been shown in animal models of Alzheimer’s disease (4), vascular dementia (5), and traumatic brain injury (6). Moreover, evidence that the glymphatic pathway is present in humans has been obtained via the intrathecal injection of MRI tracers, with delayed tracer clearance in a cohort of patients with dementia (7).

However, the glymphatic hypothesis has proven to be highly controversial (8, 9), particularly regarding the proposed bulk flow of ISF (10–12) and the mediating role of AQP4 (8, 13, 14). A key limitation of previous studies has been the use of tracer molecules that are much larger than water (18 Da) — for example, in ex vivo fluorescence imaging (700–3000 Da) (1) and in vivo MRI (Gd-DTPA, 938 Da) (15). The fact that these tracers cannot be transported by AQP4 channels most likely means that they underestimate the true magnitude of ISF flow, which will depend on the rate at which CSF water molecules enter the interstitium. We reasoned that using water molecules as a tracer would yield insights into glymphatic transport and the role of AQP4 channels.

Water molecules have previously been used as tracers by exploiting various isotopes. For example, tritiated water (^3^H_2_O) has been used to investigate CSF transport by measuring the radiation levels of blood samples using a liquid scintillation counter (16). H_2_^15^O has been imaged with positron emission tomography (17), though its short half-life (122.2 seconds) makes it impractical for studying glymphatic function. The stable ^17^O isotope is not radioactive but possesses a quadrupolar nucleus (I = 5/2) that can be imaged using MRI. Although direct imaging of the ^17^O signal is difficult due to its low gyromagnetic ratio and low natural abundance (0.037%) (18), the presence of H_2_^17^O can be detected indirectly via the effect of the quadrupolar ^17^O nucleus on the ^1^H MRI signal (19), where the quadrupolar ^17^O nucleus reduces the T2 relaxation times of bonded hydrogen nuclei. This effect is further magnified by the exchange of hydrogen atoms with other surrounding water molecules. H_2_^17^O produced from ^17^O_2_ gas has been used to image metabolism (20), and H_2_^17^O has also been administered as a tracer to image cerebral blood flow (21). In a series of studies, the Nakada group employed indirect ^1^H detection of H_2_^17^O to investigate the interstitial circulation. Using an i.v. bolus injection of H_2_^17^O (20% ^17^O enriched), they showed that exchange of blood water with the ISF and CSF was dependent on AQP4 and not AQP1 (22, 23). They also used the same method to demonstrate that water influx into CSF was significantly impaired in surfactant protein–bearing (SP-bearing) transgenic mice (24).

In order to investigate the glymphatic system more directly, we decided to infuse H_2_^17^O into the CSF at the cisterna magna, thus replicating the delivery approach used by Iliff et al. (15) in their seminal Gd-DTPA MRI experiments. To avoid elevating the intracranial pressure, the H_2_^17^O has to be infused slowly (1.8 μL/min) (25), thus limiting the amount that can be delivered. To make possible the detection of small ^1^H MRI signal changes, it was necessary to use the highest available enrichment of H_2_^17^O (90% ^17^O enriched) combined with a high signal-to-noise ratio (SNR) T2-weighted imaging sequence (SNR > 200).

## Results

**Figure 1:**
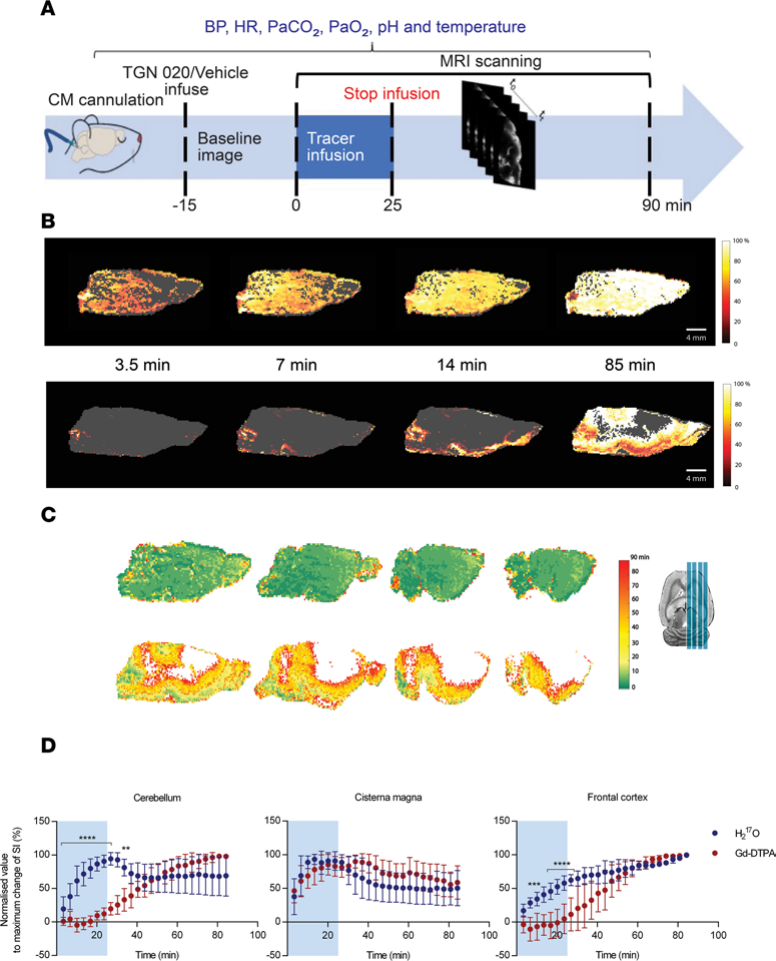
Serial MRI using H_2_^17^O tracer reveals rapid glymphatic flow over whole brain. (**A**) Study design. Rats received infusion of tracer via cisterna magna. Baseline MRI was acquired, followed by infusion of tracer and continuous MRI. (**B**) Representative sagittal MRI demonstrating the temporal evolution of tracer over 85 minutes of recording. Normalized pseudocolor scaling illustrates tracer distribution of the 90% ^17^O-enriched water (MW, 19 Da) (upper panel) and the paramagnetic tracer Gd-DTPA (Magnevist; MW, 938 Da) (bottom panel), where white in the color bar indicates maximum signal change. Representative arrival time maps. The upper panel images show a rat infused with H_2_^17^O, and the bottom panel images show 1 rat infused with Gd-DTPA. (**C**) Corresponding tracer arrival time maps for 4 sagittal slices. White color indicates that the tracer did not arrive within the 85-minute recording. (**D**) Summary data showing the normalized percentage signal change as a function of time for H_2_^17^O (*n* = 6, blue circles) and Gd-DTPA (*n* = 6, red circles) for the cerebellum, cisterna magna and frontal cortex. Blue shading on schematic drawing illustrates the location of ROIs. Blue shading on graphs indicates period of tracer infusion. *P < 0.05, **P < 0.01, ***P < 0.001, ****P < 0.0001. Data are presented as mean ± SD.

To directly image glymphatic water transport within the rat brain, the highly enriched H_2_^17^O tracer (90% ^17^O enriched) was infused into the CSF of anesthetized rats at the cisterna magna. Serial MRI revealed glymphatic transport that was strikingly more rapid and extensive than previously observed using conventional Gd-DTPA tracers (Figure 1B) (15). Movement of the Gd-DTPA tracer through the subarachnoid space was slightly slowed compared with that of H_2_^17^O. Moreover, as previously observed (15), the Gd-DTPA tracer was slow to penetrate the parenchyma, resulting in a buildup of Gd-DTPA concentration in the subarachnoid space and ventricles.

**Figure 2:**
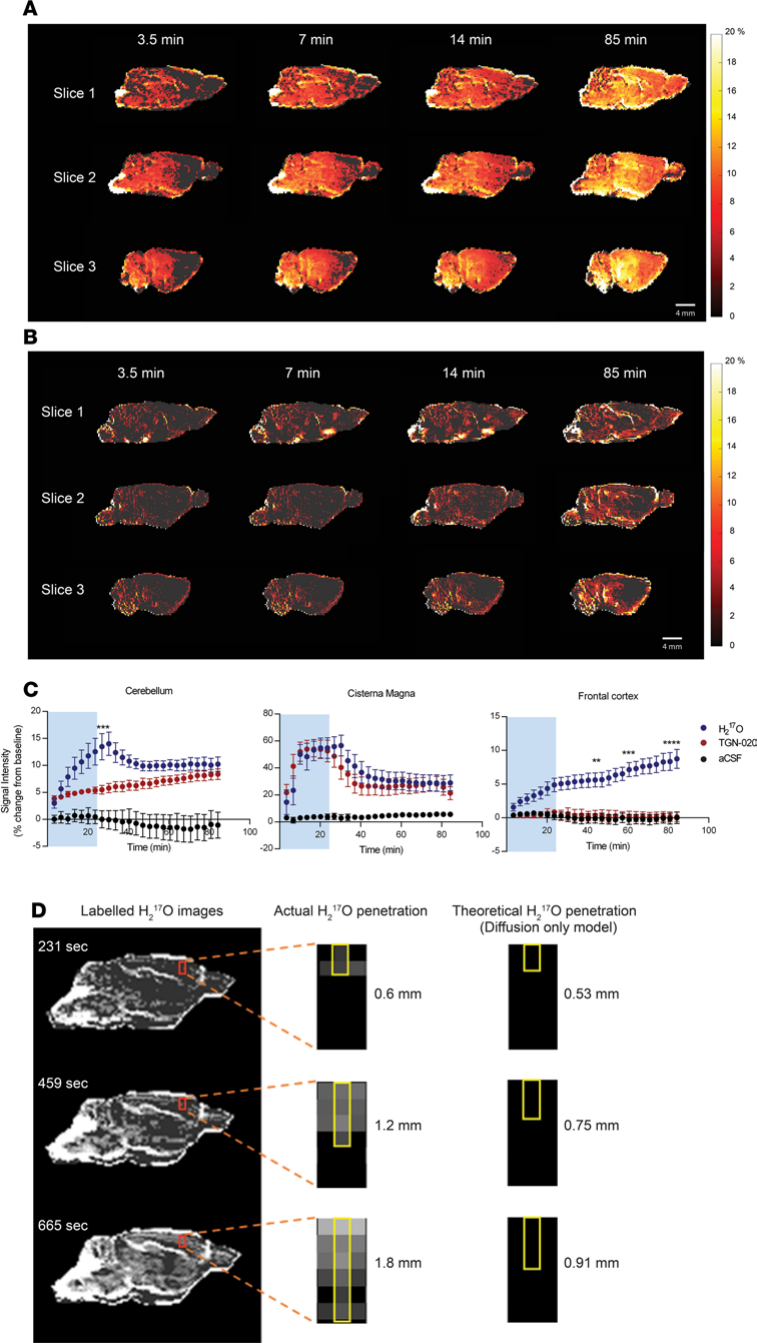
Effect of inhibiting AQP4 channels on glymphatic water flow. (**A** and **B**) Serial sagittal MRI revealing the temporal evolution of H_2_^17^O tracer for vehicle-treated rats (upper panel, *n* = 6) (**A**) and rats treated with AQP4 inhibitor (lower panel, TGN 020, *n* = 6) (**B**). Pseudocolor scaling illustrates the distribution of H_2_^17^O throughout the brain over 80 minutes of recording, with AQP4 inhibition resulting in substantially reduced H_2_^17^O transport compared with the vehicle. (**C**) Summary data showing the percent signal change as a function of time for the vehicle (blue circles) and AQP4 inhibitor–treated (red circles) groups in the frontal cortex, cerebellum, and cisterna magna. Artificial CSF (aCSF; i.e., without ^17^O-enriched H_2_^17^O) was used as a negative control. Blue shading on graphs indicates period of contrast agent infusion. (**D**) Representative images of H_2_^17^O transport at different time points. The red rectangle has been magnified to better show the actual penetration of H_2_^17^O, with the top of the red rectangle position on the cerebral cortex. The theoretically calculated displacements of H_2_^17^O due to diffusion only are 0.53 mm, 0.75 mm, and 0.91 mm at times 231, 459, and 665 seconds, respectively. It is clear that the rapid H_2_^17^O penetration of the parenchyma cannot be explained by diffusion alone, indicating the presence of convective ISF flow. *P < 0.05, **P < 0.01, ***P < 0.001, ****P < 0.0001. Data are presented as mean ± SEM.

By contrast to the Gd-DTPA tracer, H_2_^17^O rapidly penetrates the parenchyma in all brain regions (Figure 1, C and D). Thus, unlike Gd-DTPA, the concentration of H_2_^17^O does not build up in the subarachnoid space and ventricles (Figure 1C). This is best appreciated by viewing Supplemental Video 1 (supplemental material available online with this article; https://doi.org/10.1172/jci.insight.141159DS1) that shows, side-by-side, the temporal evolution of both tracers. For example, H_2_^17^O fully penetrates the rostral cortex within 10 minutes (Figure 1C), whereas, as previously observed (15), Gd-DTPA does not penetrate even after 85 minutes. There is much debate in the literature regarding the existence (9), or lack thereof (8), of a bulk convective flow of ISF. From previous measurements, we estimate, using equation 7 (below), the root mean squared (rms) displacement of water molecules in the brain to be 1.05 mm in 15 minutes, whereas — for Gd-DTPA molecules — it is 0.68 mm. This reflects the larger molecular weight of Gd-DTPA (938 Da) compared with H_2_^17^O (19 Da). In addition, Gd-DTPA molecules are restricted to the extracellular compartment, whereas H_2_^17^O is able to more freely diffuse into cells. However, comparing the estimated water displacements due to only diffusion, with the actual H_2_^17^O images (Figure 2D), it appears unlikely that diffusion alone explains the rapid brain-wide distribution of H_2_^17^O after 15 minutes, supporting the argument for a bulk convective flow of the ISF.

We next exploited the H_2_^17^O tracer to investigate the importance of AQP4 channels to glymphatic transport. The group pretreated with an AQP4 inhibitor (TGN-020, i.p., IC_50_ = 3.1 μM) (26) experienced an 80% ± 10% reduction in H_2_^17^O transported into the parenchyma compared with the vehicle group (Figure 2, A and B). It is clear that, as the AQP4 inhibitor prevents H_2_^17^O penetrating the parenchyma, the concentration of H_2_^17^O builds up in the subarachnoid space and ventricles. Indeed, the distribution of H_2_^17^O in the brains of the AQP4 inhibited group (Figure 2B) resembles the distribution of the Gd-DTPA tracer in the control group (Figure 1B).

The distribution of both tracers within the cerebellum is particularly interesting. In the control group, the Gd-DTPA tracer was slow to penetrate the cerebellum, but by 85 minutes after injection, it did penetrate the outer regions (Figure 1, B and D). However, H_2_^17^O quickly penetrated the whole cerebellum but was more rapidly removed from the white matter (Figure 1B). In the group pretreated with an AQP4 inhibitor (TGN-020), the penetration of H_2_^17^O into the gray matter of the cerebellum was considerably slowed but was strong by 85 minutes, whereas there was still no penetration of the white matter even after 85 minutes. This is presumably related to the high concentration of AQP4 in the cerebellum (27).

## Discussion

A serious limitation of previous in vivo glymphatic experiments has been their use of large tracer molecules (MRI tracer Gd-DTPA, MW 938 Da) that cannot be transported by AQP4 water channels in the brain. We hypothesized that the use of these large tracer molecules would result in the underestimation of the rate that CSF water molecules enter the brain parenchyma, thereby underestimating the true flow of interstitial water. By labeling actual water molecules, via highly ^17^O enriched water, we were able to detect glymphatic water transport in vivo using high SNR T2-weighted MRI. The ability of our H_2_^17^O MRI method to directly image water transport in vivo provides an accurate means of studying glymphatic transport.

Reasons for the discrepancy between the transport of H_2_^17^O and Gd-DTPA tracers include the large difference in molecular weight (19 Da for H_2_^17^O and 938 Da for Gd-DTPA) and the presence of the astrocytic water channel AQP4. Molecular tracers such as Gd-DTPA that lack a specific transport pathway (such as ion transporters or channels) are able to reach the parenchyma only through the ~20 nm clefts between overlapping astrocytic end feet. Water, however, is also able to travel through the ICS mediated by highly selected AQP4 water channels, which are expressed in astrocytic end feet that cover the entire vasculature of the CNS. For this reason, it is likely that previous experimental studies based on large tracer molecules may have systematically underestimated subarachnoid CSF water flow into the brain and, thus, underestimated the convective bulk flow of ISF. Asgari et al. (28) modeled the astrocytic syncytium between CSF and the compartment of the brain interstitium by including, in their model, AQP4 on the plasma membranes, an abundance of AQP4 on the perivascular surfaces, and 20 nm inter–end feet gaps. They demonstrated that the resistance to water flow through extracellular space (ECS) is 2 orders of magnitude larger than through the intracellular space (ICS) of astrocytes. This appears to be a likely explanation for the rapid penetration of the parenchymal that is seen using the H_2_^17^O tracer compared with Gd-DTPA.

An alternative hypothesis of CSF physiology has been proposed Oreskovic and Klarica (29), in which CSF production and absorption occurs at the level of the capillaries and depends on hydrostatic and osmotic forces. That hypothesis was not supported by our experiments using an AQP4 inhibitor (TGN-020); our experiments clearly demonstrated that the rapid penetration of H_2_^17^O into the parenchyma (Figure 2) is strongly dependent on AQP4, which is absent from the endothelium of brain capillaries. However, it does support the glymphatic hypothesis of Iliff et al. (15).

Klarica et al. have also suggested a continuous bidirectional mixing of water between the blood, ISF, and the CSF compartments, with no unidirectional net flow of CSF along the CSF spaces (30). Again, this is not supported by our H_2_^17^O experiments (Figure 1B). Infusing H_2_^17^O into the cisterna magna leads to a high uptake in the subarachnoid spaces (Figure 2A), and this implies that H_2_^17^O flows largely through the CSF compartment and subarachnoid space. Furthermore, the H_2_^17^O signal change was greatest along the ventral brain surface and along the margins of the pineal recess and the olfactory bulb. These anatomical structures were previously identified by Iliff et al. as the proximal glymphatic transport pathway for CSF within the wider subarachnoid space (15). Again, these observations provide support for the glymphatic hypothesis of Iliff et al. (15).

Similar to natural sleep, general anesthesia has been shown to enhance the transport of CSF tracers (e.g., Gd-DTPA). Studies by the Nedergaard group reported CSF tracer transport was highest under ketamine/xylazine (K/X) anesthesia and lower with α-chloralose, Avertin, or isoflurane (31). At least 1 of the effects of anesthesia appears to be increased extracellular volume fraction (32), which may explain the enhanced transport of extracellular tracers. However, as the H_2_^17^O tracer can more freely travel through intracellular and extracellular space, we would hypothesize that sleep or general anesthesia may have less influence on its rate of transport.

In summary, we have conclusively demonstrated that glymphatic flow imaged using our H_2_^17^O tracer is much more rapid and extensive than when imaged using the Gd-DTPA tracer (Supplemental Video 1). This is strong evidence that the ISF experiences a substantial bulk flow, which can more rapidly clear waste molecules from the parenchyma compared with diffusion alone. Furthermore, we were able to conclusively demonstrate that these glymphatic flows are strongly mediated by AQP4. We believe these advances will not only answer much of the controversy surrounding the glymphatic hypothesis, but will also provide a valuable tool for future investigations into associated neurological disorders.

## Methods

### Study design.

Male Wistar rats (280–300 g, 20–24 weeks old) were obtained from Charles River Laboratories and were randomly assigned to 5 experimental groups. The first study was designed to test our hypothesis that the use of the H_2_^17^O tracer would provide a more accurate measurement of glymphatic transport than Gd-DTPA. Two groups were used (H_2_^17^O [*n* = 7], Gd-DTPA [*n* = 7]). The second study was designed to test the hypothesis that AQP4 channels are critical to glymphatic transport using our H_2_^17^O method (AQP4 inhibitor [*n* = 6], vehicle [*n* = 6], artificial CSF [aCSF; *n* = 3]). After the surgical implantation of an intracisternal cannula, each rat was placed inside the MRI scanner, and MRI was continued for a total of 85 minutes (Figure 1A). In the drug-treated group, TGN-020 was administered i.p. 15 minutes prior to the MRI study. Respiration, blood pressure (BP), and heart rate (HR) were continuously monitored during MRI measurements, and body temperature was maintained at 37.0°C ± 0.5°C (Supplemental Table 1).

### Supplemental video.

The video displays differences in the brain-wide distribution of H_2_^17^O (90% ^17^O enriched water) and Gd-DTPA (Magnevist, 21 mM) over 85 minutes of recording. The influx of the H_2_^17^O tracer was faster and much more extensive across the whole brain.

### Surgery and physiological monitoring.

All animals were initially anesthetized (5% isoflurane in 30:70 O_2_/NO_2_ mixture) in an induction chamber, intubated, and artificially ventilated (with 2%–3% isoflurane in 30:70 O_2_/NO_2_ mixture). Body temperature was monitored throughout the experiment, with a rectal thermocouple and maintained at 37°C ± 0.5°C. The femoral artery was cannulated with PE-50 tubing for continuous monitoring of the mean arterial BP and HR (Biopac Systems, MP100) and for the measurement of arterial blood gases (Bayer, Rapidlab 248). The animal was then transferred to a stereotaxic frame, where the head was secured with ear and tooth bars. The head was tilted (45°, snout down), and a midline skin incision was made to expose the dura mater overlying the cisterna magna space. A CM cannula (22-gauge PEEK, SAI, RCMC-01) connected to a closed-end PE10 tube loaded with aCSF (NaCl 140 mmol/L, KCl 3 mmol/L, NaH2PO4 12 mmol/L, NaHCO3 18 mmol/L, CaCl2 2.5 mmol/L, pH 7.4) was advanced 2 mm into the intrathecal space and secured with cyanoacrylate glue to avoid leakage. In the drug treatment group, 200 mg/kg TGN-020 (MilliporeSigma), dissolved in DMSO (10 mM) solution, was administered i.p. (200 mg/kg in 5 mL) 15 minutes before starting the MRI study. Animals were placed prone in a cradle, transferred to the MRI scanner, and monitored and maintained under anesthesia within the physiological range. The CM cannula was connected to a length of PE10 tubing (2.5 cm long, 0.28 mm ID × 0.61 mm OD, Braintree Scientific) filled with the desired tracer (13.5 mM Gd-DOTA, 90% H_2_^17^O or aCSF) and attached to an infusion pump (Graseby 3150 Syringe Pump) for tracer delivery. The infusion pump was evaluated for accuracy by measuring the mass of a target volume of a tracer delivered at the determined flow rate to account for losses and/or bubbles in the infusion system. Respiration, BP, and HR were continuously monitored during MRI measurements, and body temperature was maintained at 37.0°C ± 0.8°C (Supplemental Table 1). At the end of the experiment, the animal was euthanized.

### MRI.

MRI data were acquired using a Bruker PharmaScan 7T/16 cm system controlled by Paravision 5.1 software (Bruker BioSpin) with a gradient coil insert (internal diameter = 90 mm, 300 mT/m) and a 4-channel phased-array surface receive coil used for rat brain imaging. Two different tracers were used for this experiment: Gd-DTPA (Magnevist, 21 mM; MW, 938 Da; Bayer HealthCare Pharmaceuticals Inc.) and H_2_^17^O (90% ^17^O-enriched water, NUKEM Isotopes). Furthermore, aCSF was used as a negative control. The scanning protocol for all studies consisted of 3 baseline scans, followed by the intrathecal infusion of tracer via the CM catheter (50 μL at 1.8 μL/min; total time, 28 min). MRI data were continually acquired over a period of 85 minutes.

### Gd-DTPA tracer imaged with T1-weighted imaging.

To visualize the glymphatic pathways using gadolinium (T1W shortening effects), 3D T1-weighted FLASH images were acquired in the sagittal plane (repetition time/echo time [TR/TE] = 15 ms/3 ms; flip angle = 15°; number of averages [NA] = 1; field of view [FOV] = 3.0 × 3.0 × 3.0 cm; total scan time = 3 minutes, 5 seconds; acquisition matrix size = 128 × 128, yielding an original image resolution of 0.234 × 0.234 × 0.234 mm). A baseline T1-weighted image was acquired before infusion, which was subtracted from the corresponding images after the infusion of Gd-DTPA. The resulting difference image reflected the distribution of Gd-DTPA in the brain. aCSF was used as a negative control.

### H_2_^17^O tracer imaged with high-SNR T2-weighted imaging.

The quadrupolar moment of the ^17^O nucleus reduced the transverse relaxation time, T2, of bonded ^1^H nuclei within a water molecule via scalar coupling. This effect was further magnified by the rapid exchange of surrounding ^1^H^+^ ions with H_2_^17^O molecules,

 (Equation 1)
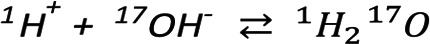


According to Meiboom (19), for low concentrations of H_2_^17^O in H_2_^16^O, the resulting ^1^H transverse relaxation time of water is given by,

 (Equation 2)
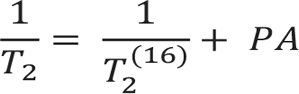


Where

 (Equation 3)
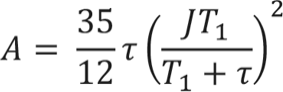


Where P is the fraction of water molecules containing ^17^O, τ is the proton exchange lifetime in H_2_^17^O, J is the scalar coupling constant for ^1^H-^17^O bond, T_1_ is the ^1^H longitudinal relaxation time, and τ is the ^1^H exchange lifetime. Hence, the presence of H_2_^17^O water can be detected by a reduction in the ^1^H signal on T2-weighted MRI. A key aspect of the MRI sequence employed here was the use of high SNR (>200) T2-weighted MRI scans to enable the detection of small signal changes. A baseline T2-weighted image was acquired before infusion. This was subtracted from corresponding images after the infusion of H_2_^17^O or aCSF (negative control), and the resulting difference image reflected the distribution of H_2_^17^O in the observed brain. T2-weighted images were acquired with a fast spin-echo sequence (rapid acquisition with relaxation enhancement [RARE]) (8 slices, slice thickness = 1.5mm, TR/TE = 3,000 ms/61 ms; NA = 4; FOV = 6.0 × 6.0 × 1.2 cm; total scan time = 3 minutes, 40 seconds; acquisition matrix size = 200 × 200, yielding an original image resolution of 0.3 × 0.3 × 1.5 mm).

### MRI data analysis.

MATLAB R2018b (MathWorks) code was developed in-house for postprocessing MRI images. The general postprocessing procedure consisted of brain extraction, head motion correction, and voxel-by-voxel conversion to a percentage signal change. Briefly, a brain mask was created for the removal of nonbrain tissue, improving the performance of the following: using rigid body alignment of each scan to the mean precontrast image, scan-to-scan misregistration caused by head movement was corrected. The resulting registrations were visually inspected to ensure adequate alignment. To ensure that voxel intensity represented a percentage change relative to the average baseline images, all time-series images were subtracted and divided by the baseline average image using the following expression:

For the Gd T1W image study:

 (Equation 4)
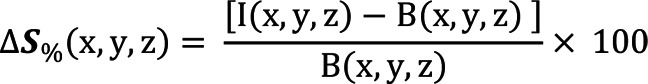


For the H_2_^17^O/aCSF T2W image study:

 (Equation 5)
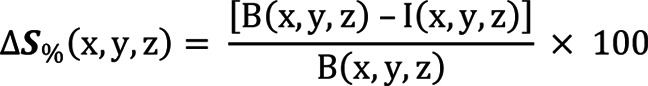


Where ΔS_%_ is the percent signal change from the baseline (B), I is the time-series image after tracer infusion, and (x,y,z) is the voxel position. The T1-weighted and T2-weighted averaged baseline images were used to anatomically guide the placement of regions of interest (ROIs). In order to compare the T2-weighted signal changes produced by H_2_^17^O with the T1-weighted signal changes produced by Gd-DTPA, both were normalized to the maximum signal change observed over the entire measurement period. Maps of the tracer arrival time, which are defined as the time that the tracer arrives at each voxel after injection, were created on a voxel-by-voxel basis.

The 1-dimensional Einstein diffusion equation can be solved to give the probability density function, *P(x,t)*, for particles undergoing Brownian motion:

 (Equation 6)
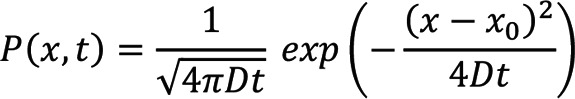


Where D is the diffusion coefficient and *x* is the particle displacement from its initial position *x_0_*, during an observation time, t. For this Gaussian function the root mean square displacement, *x_rms_*, of an ensemble of particles is given by:

 (Equation 7)
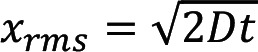


From previous diffusion MRI experiments using a long observation time (600 ms) (33), we measured the plateau diffusion coefficient of water within the rodent cortex as 0.00062 mm^2^/s. From equation 7, the root mean square displacement, x_rms_, of water for experiment times of 3.85 minutes (231 sec), 7.65 minutes (459 sec), 11.08 minutes (665 sec), and 14.83 minutes (893 sec) was calculated to be 0.53 mm, 0.75 mm, 0.91 mm, and 1.05 mm, respectively. Previous phantom studies of Gd-DTPA diffusivity in a tissue mimic material (polyvinyl alcohol-cryogel) have been performed using MRI, giving a diffusion coefficient of 0.00026 mm^2^/s (34). From equation 2, the root mean square displacement, x_rms_, of Gd-DTPA for experiment times of 7.65 minutes (459 sec), 11.08 minutes (665 sec), and 14.83 minutes (893 sec) were calculated to be 0.49 mm,0.59 mm, and 0.68 mm, respectively.

### Statistics.

No statistical methods were used to predetermine sample size, with sample sizes being similar to those reported in a previous study (15). Statistical tests performed using GraphPad Prism (GraphPad Prism Software). Statistical comparisons between groups were performed by repeated measures 2-way ANOVA, followed by Sidak’s test to correct for multiple comparisons. All tests were considered statistically significant for *P* < 0.05. All data are presented as mean ± SD unless otherwise stated.

### Study approval.

Experiments were carried out under license from the UK Home Office in accordance with the Animals (Scientific Procedures) Act, 1986, incorporating European Directive 2010/63/EU and approved by the University of Glasgow Ethical Review Panel.

### Author contributions.

MSA and WMH developed the MRI method, contributed to conceiving and designing experiments, analyzed all data, produced the figures, and contributed to discussion and interpretation of results and the writing of the final manuscript. LG contributed to the design of the experiments, performing surgical procedures on rats and acquiring the MRI data. LG also contributed to writing the manuscript. LMW was the secondary Ph.D. supervisor of MSA and contributed to the design of the experiments, to discussion and interpretation of results, and to the writing of the final manuscript.

## Supplementary Material

Supplemental data

Supplemental Video 1

## Version 1 04/15/2021

In-Press Preview

## Version 2 05/24/2021

Electronic publication
