# Supplementary material for: Direct imaging of glymphatic transport using H217O MRI
Source: JCI Insight. 2021 May 24;6(10):e141159. doi: 10.1172/jci.insight.141159 (PMC8262348; doi:10.1172/jci.insight.141159)
Supplement: Supplemental data [file jciinsight-6-141159-s028.pdf]

Supplemental Table 1. Physiological parameters of all experimental groups.

Physiological parameters		Pre- infusion	Post- infusion
PaO₂ **(mmHg)**	$H_2{}^{17}O$	102.1 ± 25.7	125.6 ± 9.6
	Gd-DTPA	94.3 ± 22.1	98.7 ± 24
	$H_2{}^{17}O$ (TGN 020)	119.3 ±16	127.9 ± 19.2
	$H_2{}^{17}O$ (Vehicle)	157.8 ± 32	180.3 ± 40
	aCSF	142.5 ± 67.4	161.9 ± 37.2
PaCO₂ **(mmHg)**	$H_2{}^{17}O$	37.5 ± 9	42 ± 9.2
	Gd-DTPA	40.7 ± 10.1	41.3 ± 8.9
	$H_2{}^{17}O$ (TGN 020)	35.4 ± 5.2	37.2 ± 9.2
	$H_2{}^{17}O$ (Vehicle)	33.4 ± 9.3	24.7 ± 8
	aCSF	49.1 ± 25.1	47.7 ± 23.5
**Temp (°C)**	$H_2{}^{17}O$	37 ± 0.6	37.2 ± 0.5
	Gd-DTPA	36.9 ± 0.7	36.7 ± 0.8
	$H_2{}^{17}O$ (TGN 020)	36.8 ± 0·5	36·9 ± 0·5
	$H_2{}^{17}O$ (Vehicle)	36.8 ± 0.7	37 ± 0.2
	aCSF	37·2 ± 0·6	37·1 ± 0·5
**pH**	$H_2{}^{17}O$	7.5 ± 0.06	7.5 ± 0.08
	Gd-DTPA	7.5 ± 0.07	7.4 ± 0.09
	$H_2{}^{17}O$ (TGN 020)	7.5 ± 0.04	7.5 ± 0.08
	$H_2{}^{17}O$ (Vehicle)	7.5 ± 0.05	7.5 ± 0.04
	aCSF	7.5 ± 0.09	7.5 ± 0.1

Abbreviations: $PaCO_2$, partial pressure of carbon dioxide; PaO_2, partial pressure of oxygen; Temp, temperature. Significant differences denoted by $P < 0.05$. Data presented as mean ± SD.
